# AttnTAP: A Dual-input Framework Incorporating the Attention Mechanism for Accurately Predicting TCR-peptide Binding

**DOI:** 10.3389/fgene.2022.942491

**Published:** 2022-08-22

**Authors:** Ying Xu, Xinyang Qian, Yao Tong, Fan Li, Ke Wang, Xuanping Zhang, Tao Liu, Jiayin Wang

**Affiliations:** ^1^ Department of Computer Science and Technology, School of Electronic and Information Engineering, Xi’an Jiaotong University, Xi’an, China; ^2^ Geneplus Beijing Institute, Beijing, China

**Keywords:** T-cell receptor, TCR-peptide binding prediction, deep learning framework, BiLSTM model, attention mechanism

## Abstract

T-cell receptors (TCRs) are formed by random recombination of genomic precursor elements, some of which mediate the recognition of cancer-associated antigens. Due to the complicated process of T-cell immune response and limited biological empirical evidence, the practical strategy for identifying TCRs and their recognized peptides is the computational prediction from population and/or individual TCR repertoires. In recent years, several machine/deep learning-based approaches have been proposed for TCR-peptide binding prediction. However, the predictive performances of these methods can be further improved by overcoming several significant flaws in neural network design. The interrelationship between amino acids in TCRs is critical for TCR antigen recognition, which was not properly considered by the existing methods. They also did not pay more attention to the amino acids that play a significant role in antigen-binding specificity. Moreover, complex networks tended to increase the risk of overfitting and computational costs. In this study, we developed a dual-input deep learning framework, named AttnTAP, to improve the TCR-peptide binding prediction. It used the bi-directional long short-term memory model for robust feature extraction of TCR sequences, which considered the interrelationships between amino acids and their precursors and postcursors. We also introduced the attention mechanism to give amino acids different weights and pay more attention to the contributing ones. In addition, we used the multilayer perceptron model instead of complex networks to extract peptide features to reduce overfitting and computational costs. AttnTAP achieved high areas under the curves (AUCs) in TCR-peptide binding prediction on both balanced and unbalanced datasets (higher than 0.838 on McPAS-TCR and 0.908 on VDJdb). Furthermore, it had the highest average AUCs in TPP-I and TPP-II tasks compared with the other five popular models (TPP-I: 0.84 on McPAS-TCR and 0.894 on VDJdb; TPP-II: 0.837 on McPAS-TCR and 0.893 on VDJdb). In conclusion, AttnTAP is a reasonable and practical framework for predicting TCR-peptide binding, which can accelerate identifying neoantigens and activated T cells for immunotherapy to meet urgent clinical needs.

## 1 Introduction

T-cell receptor (TCR) hypervariable regions are formed by complex recombination of genomic precursor elements that mediate recognition of antigens presented by peptide-major histocompatibility complex (pMHC) molecules (La Gruta et al., 2018; Joglekar and Li, 2021). Complementary determining region 3 (CDR3) is the key structural feature located within the TCR variable regions, and specific CDR3-pMHC complexes enable T cells to recognize and eliminate evolving pathogens or malignant cells (La Gruta et al., 2018; Joglekar and Li, 2021). Thus, the CDR3 region, derived from quasi-random mutations of V(D)J recombination, is considered to have a primary function in recognizing the endogenous and exogenous antigens in the immune-dominant T-cell process and resulting “TCR repertoire” in an individual, which defines a unique footprint of cellular immune protection (Chiffelle et al., 2020).

The high-throughput immune repertoire sequencing (IR-seq) can capture millions of sequencing reads derived from the hypervariable regions and produce detailed T-cell repertoires for individual or population analysis, such as epitope prediction (Warren et al., 2011; Woodsworth et al., 2013; Glanville et al., 2017). However, identifying epitopes from TCR repertoires by biomechanical experiments is a time-consuming and labor-intensive task. An epitope that is expanded in multiple T-cell clones is more likely to be exposed to the pMHC complex and can generally serve as a surface biomarker for immunotherapy or vaccine targets. Fortunately, the availability of immune-related TCR/BCR sequence databases, such as IEDB (Mahajan et al., 2018), VDJdb (Bagaev et al., 2020), and/or McPAS-TCR (Tickotsky et al., 2017), will serve as motivation to accelerate the development of well-integrated epitope prediction pipelines. As a result, it will be an ideal method that predicts an epitope from billions of TCR sequences and validates it with a biological experiment, greatly reducing time and cost consumption.

It is critical to introduce an appropriate prediction model to predict an epitope, as extracting fitness features from a highly variable and shortened amino acid chain is difficult (Bolotin et al., 2012). The length and positional characteristics of the subsequences are unknown, and the amino acids in the subsequences contribute to varying degrees. Unfortunately, the aforementioned public databases have an imbalanced epitope distribution (a high number of unseen epitopes) as well as a lack of high-quality labeled seen-epitope data (Moris et al., 2021). Deep machine learning (DL) models have significantly accelerated the epitope prediction task by automatically learning engineering features based on domain knowledge and extracting unknown and implicit features from unprecedented amounts of TCR repertoire data using unprecedented scale models (LeCun et al., 2015; Zemouri et al., 2019; Tran et al., 2022).

Several cutting-edge TCR-peptide binding prediction approaches based on DL frameworks have been proposed in the last 2 years, and they were applicable to both seen and unseen-TCR epitopes. DLpTCR used a multi-model ensemble strategy comprised of three base classifiers in predicting the likelihood of interaction between TCR αβ chains and peptides (Xu et al., 2021). NetTCR-2.0 provided a 1-dimensional (1D) convolution neural network (CNN) architecture combining max-pooling for dealing with sequence length variations (Montemurro et al., 2021). The input TCR αβ chains and peptide sequences were encoded by the BLOSUM50 (Henikoff and Henikoff, 1992) matrix before being fed into a dense layer for prediction. ImRex used a four-layer convolution and two-layer max-pooling CNN architecture to predict the combined representation of CDR3 and peptide sequences, by extracting their physicochemical properties as features (Moris et al., 2021). ERGO employed a new multilayer perceptron (MLP) model to predict the likelihood of TCR-peptide binding. During the study, they provided two different encoding methods, a long short-term memory (LSTM) network, and an auto-encoder network to generate the corresponding models (ERGO-LSTM & ERGO-AE) (Springer et al., 2020).

The CNN architecture is widely used to extract the features of TCRs and make TCR-peptide prediction, such as DLpTCR, ImRex, NetTCR-2.0 and DeepLION (Xu et al., 2022), due to its superior capacity for image feature learning. However, the lack of CNN memory capability during the model process will reduce the feature extraction performance on short sequence data, especially TCRs. Due to the spatial folding of TCRs, amino acids in sequences may be related not only to their adjacent amino acids, but also to some more distant ones. When extracting sequence features, CNN only considered interrelationships between adjacent amino acids and ignored those between non-adjacent amino acids, which also play a significant role in TCR antigen-binding specificity. The LSTM architecture, used by the ERGO model, had memory capability and would reduce the information loss of non-adjacent amino acids. However, the ERGO model only used the last node output to represent the entire sequence, ignoring the contribution of previous node outputs to the final prediction. Furthermore, the existed start-of-art models could not pay more attention to the amino acids in sequences that contributed significantly to TCR antigen recognition. The complex framework would result in overfitting on TCR-peptide binding tasks, especially under unbalanced datasets with small labeled sample sizes. As a result, there were still some unresolved issues with existed models and their predictive performances can be further improved by overcoming several significant flaws in neural network designs.

**FIGURE 1:**
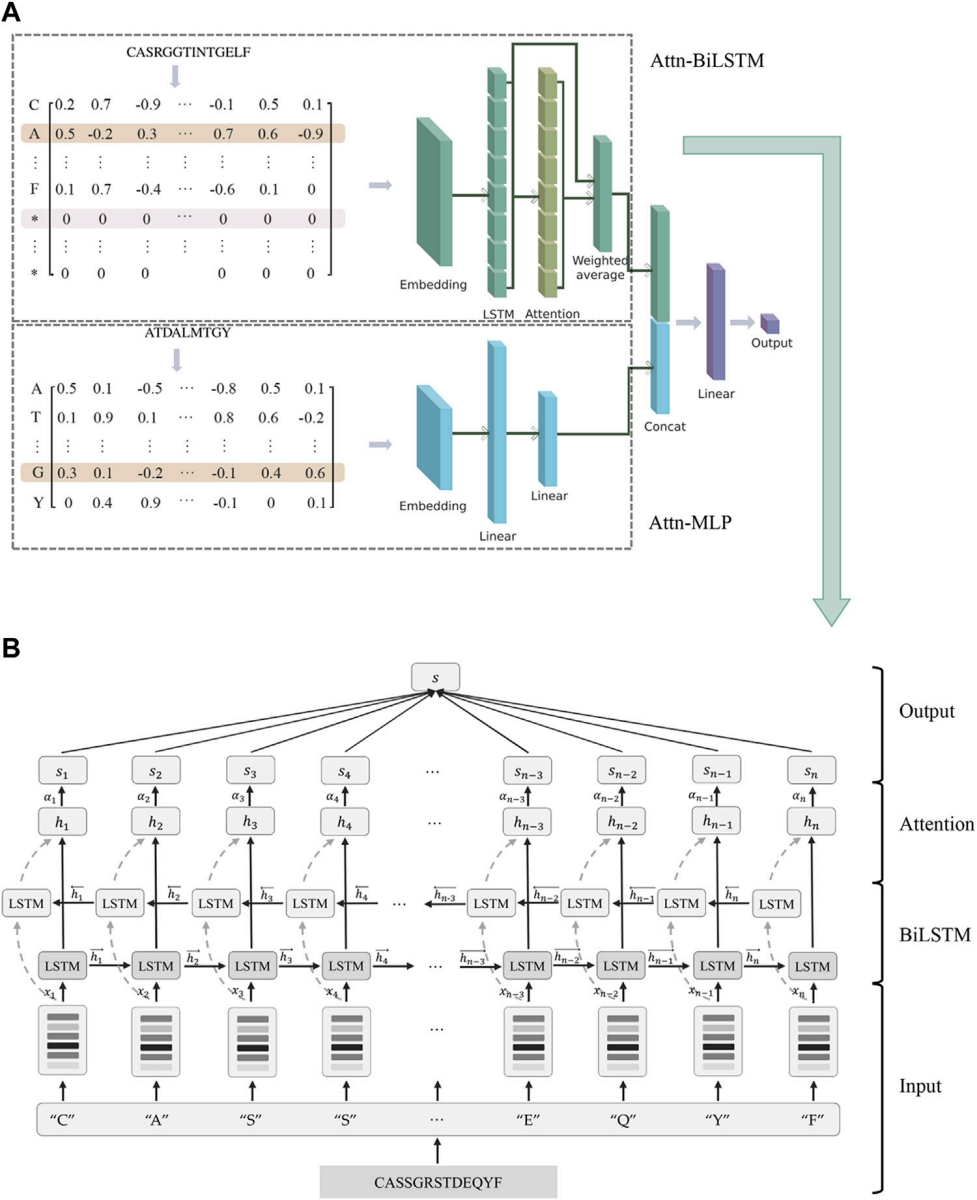
AttnTAP improved the prediction accuracy of TCR-peptide binding. **(A)** AttnTAP was a dual-input deep learning framework, which included the feature extractors for TCR and peptide sequences, Attn-BiLSTM and Attn-MLP. The corresponding feature vectors extracted by the two models were then concatenated for predicting the likelihood of TCR and peptide binding using the multilayer perceptron network. **(B)** The feature extractor for TCR sequences, Attn-BiLSTM, was divided into four parts: the input layer, bi-directional long short term memory (BiLSTM) layer, attention layer, and output layer. Sequences were preprocessed and encoded into embeddings in the input layer. The embeddings were then fed into the BiLSTM and attention layers, respectively. The BiLSTM layer extracted the sequences’ feature vectors, while the attention layer computed the weights of each position in the sequences. Finally, the output layer outputted the weighted feature vectors.

Motivated by these, we proposed AttnTAP, a dual-input deep learning network that included the Attn-BiLSTM and Attn-MLP models, to improve the prediction of TCR-peptide binding (Figure 1). The bi-directional LSTM (BiLSTM) model with an attention mechanism was used to extract the features of TCR sequences, as described in Section 2.2. The BiLSTM model considered the interrelationships between amino acids and their adjacent or non-adjacent precursors and postcursors. Moreover, due to the attention mechanism, all node outputs were used to represent the entire sequence after weighted calculation, with a focus on the key amino acids. Given that very few known peptides in the public databases compared to the TCR sequences, a simple network, MLP, was used to extract peptide features to reduce the complexity of the network structure. A dual-input framework of CDR3 sequences and peptides was used to combine embedding matrices, and then the two output feature vectors were concatenated by the MLP network to predict the likelihood of a TCR recognizing a peptide. Finally, we evaluated the performance of AttnTAP and other start-of-art TCR-peptide binding prediction models, in terms of the prediction accuracy, computational cost, and space complexity.

## 2 Materials and methods

AttnTAP was a dual-input deep learning framework developed for predicting the TCR-peptide binding (Figure 1A). TCR CDR3β sequences, as one of the inputs, were extracted features using the BiLSTM model with an attention mechanism, named Attn-BiLSTM. The peptide sequences were extracted features using the MLP model, named Attn-MLP. Then, the corresponding features from Attn-BiLSTM and Attn-MLP models were concatenated to form a final feature that was used to predict the likelihood of TCR-peptide binding using the MLP network.

### 2.1 Data processing

The public TCR-peptide datasets used in this study were downloaded from the VDJdb (https://vdjdb.cdr3.net/) (Bagaev et al., 2020), IEDB (http://www.iedb.org/) (Mahajan et al., 2018), and McPAS-TCR (http://friedmanlab.weizmann.ac.il/McPAS-TCR/) (Tickotsky et al., 2017), respectively. The three datasets were used to train the word vectors for AttnTAP, and the VDJdb and McPAS-TCR datasets were used to evaluate the performance of binding prediction approaches. In all of the three datasets, the standard screening sequences are as follows: 1) We removed the duplicated sequences, too short (<6bp) or too long (>30bp) CDR3β sequences, incomplete sequences, and tag-less sequences; 2) The peptide sequences corresponding to less than 50 TCR sequences were also removed; 3) We retained only the correct sequences of the human TCRβ CDR3 and peptide sequences. As result, we obtained amounts of 181,436 CDR3β sequences from the three public datasets (“CA … F” sequences) to train the word vectors for AttnTAP (dataset one in this study). The length of CDR3β sequences ranges from 6 to 27 amino acids, with the majority containing 11–18 amino acids (Supplementary Figure S1).

Furthermore, after the screening process, we obtained 9,597 TCR-peptide pairs with 25 different peptide sequences from the McPAS-TCR database and 38,134 TCR-peptide pairs with 56 different peptide sequences from the VDJdb database as positive samples (Table 1, dataset two in this study). We analyzed these peptides in the datasets and their species, TCR counts, and abundances are shown in Supplementary Table S1. Negative samples were generated by randomly replacing the corresponding peptide in positive samples with other peptides (Springer et al., 2020). The procedure for generating negative samples is shown in Supplementary Algorithm S1. The ratio of negative samples to positive samples used in this study ranged from 1:1 to 15:1.

**TABLE 1:** The datasets used for approach evaluation.

	Peptide type	TCR-peptide pair number	Positive sample size	Negative sample size
**McPAS-TCR**	25	9,597	9,597	9,597–143,955
**VDJdb**	56	38,134	38,134	38,134–572,010

### 2.2 Attn-BiLSTM model

Attn-BiLSTM model was divided into four parts including the input layer, BiLSTM layer, attention layer, and output layer (Figure 1B). In the input layer, amino acid sequences were preprocessed and encoded into embeddings. Then, the embeddings were fed into both the BiLSTM and the attention layers. The feature vectors of sequences were extracted in the BiLSTM layer, while the weights of each position in the sequences were computed in the attention layer. Finally, the weighted feature vectors were output in the output layer.

#### 2.2.1 Input layer

According to the previous studies (Montemurro et al., 2021) and length-frequency statistics (Supplementary Figure S1), the maximum input length of CDR3 was 18 amino acids and the redundant part would be truncated to a longer sequence. For the shorter sequences, we completed them with a placeholder “X” to the maximum length.

Random initialization vectors and pre-training word vectors were available for Attn-BiLSTM to encode sequences. We used the character granularity vectors and word granularity vectors as pre-training word vectors, respectively. Each amino acid was viewed as a basic character, resulting in a total of 20 characters. Moreover, three consecutive amino acid residues in a sequence were considered as one word in word granularity vectors, also named triplet word vectors (Asgari and Mofrad, 2015). We used Word2vec (Mikolov et al., 2013) to train these word vectors.

#### 2.2.2 BiLSTM layer

The LSTM model specializes in sequential data, reduces information loss and long-term dependency problems in the recurrent neural network, and performs well in TCR-peptide binding prediction (Springer et al., 2020). Compared to the LSTM, BiLSTM allows for more comprehensive and robust feature extraction because it takes into account both precursor and successor positions (Zhou et al., 2016). As a result, the BiLSTM model was used to extract the features of CDR3 sequences in this experiment. The encoded vector in the *i*th position *x*_*i*_ was fed into the forward LSTM (from left to right) and backward LSTM (from right to left) network, and the feature vectors $\vec{h_{i}}$ and $\overset{\leftarrow }{h_{i}}$ were output, respectively.

#### 2.2.3 Attention mechanism

**FIGURE 2:**
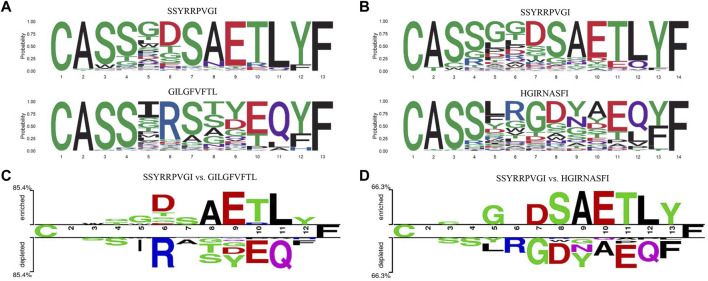
Amino acid composition of CDR3 sequences bound to different peptide. **(A,B)** The seqlogo graphs of CDR3 sequences to different peptides. **(C,D)** The "Two Sample Logo" graphs of CDR3 sequences to two different peptides.

As an example, we plotted the seqlogo graphs of CDR3 sequences corresponding to the peptide sequences (Figures 2A,B) (Wagih, 2017), which indicated that the CDR3 sequences corresponding to different peptide sequences had similar patterns in upstream and downstream targets, but extremely distinct in the middle region. The difference between CDR3 sequences, corresponding to two different peptide sequences at various positions using “Two Sample Logo” (Figures 2C,D) (Schneider and Stephens, 2002; Crooks et al., 2004), also indicated that the amino acid composition of CDR3 sequences binding to different peptide sequences varies widely.

As shown in the aforementioned example, due to the significant differences in amino acid composition in the middle region of the CDR3 sequence, the attention mechanism could be used to focus on the amino acids that contributed to the antigen-binding specificity and improve the feature extraction (Vaswani et al., 2017; Bahdanau et al., 2014). The weight of the feature vector in the *i*th position was calculated as

$$\begin{array}{c} u_{i}=\mathrm{Tanh}\left(W_{A}h_{i}+b_{A}\right), \end{array}$$ (1)

$$\begin{array}{c} a_{i}=\frac{e^{u_{i}^{\text{T}}u}}{\sum_{t}e^{u_{t}^{\text{T}}u}\;}, \end{array}$$ (2)

where $W_{A}\;$ and $b_{A}$ were, respectively, the weight matrix and bias, Tanh(*x*) was the activation function, and *a*_*i*_ was the regularization of *u*_*i*_ using the Softmax function.

### 2.3 Attn-MLP model

Attn-MLP for peptide sequences consisted of the input layer and MLP layer. The input layer was the same as that in Attn-BiLSTM, and we set the maximum length of peptide sequences to nine in our study. We used a two-layer MLP model, a simple neural network model used in the majority of TCR-peptide binding prediction approaches (Springer et al., 2020; Montemurro et al., 2021; Moris et al., 2021; Xu et al., 2021), to extract the features of peptides. The operation process in each layer of the MLP model was given by

$$\begin{array}{c} x^{\prime }=\mathrm{ReLU}\left(W_{M}\cdot x+b_{M}\right), \end{array}$$ (3)

where $W_{M}\;$ and $b_{M}$ were, respectively, the weight matrix and bias, and ReLU(*x*) was the activation function to avoid gradient explosion or disappearance. To avoid overfitting, we used dropout (Srivastava et al., 2014) with a rate of 0.1.

### 2.4 Multilayer perceptron network

The feature vectors of TCR and peptide sequences were concatenated into a final feature vector, which was used as the input of the latter MLP network for classification. The operation process of the MLP network was similar to Eq. 3, and the final prediction output was shown as

$$\begin{array}{c} \tilde{Y}=P\left(Y=1|\left\{TCR_{i},\;{\text{Peptied}}_{j}\right\}\right)=\mathrm{ReLU}\left(W_{M}^{\prime }\cdot x^{\prime }+b_{M}^{\prime }\right), \end{array}$$ (4)

where $\tilde{Y}$ denoted the probability that the *i*th TCR sequence binds to the *j*th peptide sequence. When $\tilde{Y}>0.5$ , we considered the TCR recognized the peptide and vice versa. The dropout with a rate of 0.1was used to avoid overfitting. AttnTAP was end-to-end trainable, and the loss function was the log-likelihood function defined as

$$\begin{array}{c} L=-\left[\tilde{Y}\text{ln}\tilde{Y}+\left(1-\tilde{Y}\right)\text{ln}\left(1-\tilde{Y}\right)\right]. \end{array}$$ (5)

### 2.5 Performance evaluation approaches

We selected several state-of-the-art TCR-peptide combination prediction methods proposed in the last 2 years, which employed deep learning frameworks, to compare their performance with AttnTAP. As a result, ERGO (Springer et al., 2020), ImRex (Moris et al., 2021), DLpTCR (Xu et al., 2021), and NetTCR-2.0 (Montemurro et al., 2021) were selected for the comparison experiments (Table 2).

**TABLE 2:** The selected representative TCR-peptide binding prediction approaches.

	Predictable TCR chain(s)	Model complexity	Input length constraint	Proposed date	Availability
**ERGO-LSTM**	TCRβ	Medium	None	August 2020	https://github.com/louzounlab/ERGO /
**ERGO-AE**	TCRβ	Low	None	August 2020	https://github.com/louzounlab/ERGO /
**ImRex**	TCRβ	High	TCR: 10–20 & Epitope: 8–11	December 2020	https://github.com/pmoris/ImRex /
**DLpTCR**	TCRα&β	High	None	July 2021	https://github.com/jiangBiolab/DLpTCR /
**NetTCR-2.0**	TCRα&β	Low	TCR: 8–18 &Epitope: 9	September 2021	https://github.com/mnielLab/NetTCR-2.0 /

#### 2.5.1 Two prediction tasks used for approach validation

Two different tasks, TCR-Peptide Pairing I (TPP-I) and TCR-Peptide Pairing II (TPP-II) as described in the previous study (Springer et al., 2020), were selected to estimate the performance of the binding prediction. In the TPP-I task, all of the TCRs and peptides both belong to the training and test sets, and TCR-peptide pairs were divided into disjoint training and test sets (dataset 2). We performed five-fold cross-validation (CV) for the TPP-I task. First, we sampled the original dataset randomly and generated a new dataset (∼10,000 TCR-peptide pairs). Then, the generated dataset was randomly divided into five equal parts, four of which were used as the training set and the rest as the test set. Three-quarters of the training data were used to train the model five times independently, and the rest were used as the validation data to select the final model.

The TPP-II was similar to TPP-I, except the TCRs contained in the pairs belonging to the training set could not belong to the test set. Considering that it was difficult to divide the dataset into five equal parts as required, we conducted independent replicate experiments 30 times to perform an unbiased estimation. The generated dataset was divided into a fixed ratio, the same as the five-fold CV in TPP-I, with a 4:1 ratio of training data to test data.

#### 2.5.2 Metrics used for performance evaluation

In this study, we used the accuracy (ACC), recall (REC), precision (PRE), F1 score (F1), and area under the receiver operating characteristic curve (AUC), as the criteria for the performance evaluation of these six approaches. There were six values in these equations, including true (T), false (F), true positive (TP), true negative (TN), false positive (FP), and false-negative (FN), were used. The formulas were presented as follows:

$$\begin{array}{c} ACC=\frac{TP+TN}{P+N}=\frac{TP+TN}{TP+FN+TN+FP},\; \end{array}$$ (6)

$$\begin{array}{c} REC=\frac{TP}{P}=\frac{TP}{TP+FN}, \end{array}$$ (7)

$$\begin{array}{c} PRE=\frac{TP}{TP+FP},\;and \end{array}$$ (8)

$$\begin{array}{c} F1=2\times \frac{PRE\times REC}{PRE+REC}.\; \end{array}$$ (9)

Computational costs are always used in computer science to evaluate an algorithm. In this study, we considered the time complexity and the space complexity, which could be represented by the average running time and the required memory occupancy of the several algorithms in each model as previously described (Zhao et al., 2020).

## 3 Results

### 3.1 AttnTAP model performance

#### 3.1.1 AttnTAP performance on different encoding methods

Three pre-training word vectors, random initialization vectors, amino acid word vectors, and triplet word vectors, were tested in the Attn-BiLSTM and Attn-MLP model, to validate their effectiveness on AttnTAP classification (Table 3). The ACC and AUC were used to evaluate the performance of the three different encoding methods on the balanced McPAS-TCR and VDJdb datasets. The random initialization vectors and amino acid word vectors showed better performance on two datasets, while the triplet word vector had the worst performance. The prediction accuracies of random initialization vectors, whose computational cost was much less, were similar to those of amino acid word vectors. Thus, the random initialization vectors were used for sequence encoding to improve the prediction accuracy of AttnTAP.

**TABLE 3:** The performance of AttnTAP with different encoding methods.

	McPAS-TCR		VDJdb	
	ACC^a^	AUC	ACC	AUC
**Random initialization**	**0.788**	**0.878**	0.843	0.910
**Amino acid word vector**	0.784	0.871	**0.847**	**0.911**
**Triplet word vector**	0.616	0.678	0.827	0.878

a Abbreviations: ACC: accuracy; AUC: area under the receiver operating characteristic curve.

#### 3.1.2 AttnTAP performance on five different TCR feature extraction models

To assess the ability of the feature extraction method at predicting accuracy, we tested the five different TCR extraction methods based on the balanced McPAS-TCR and VDJdb datasets. The five different TCR feature extraction methods were (I) the MLP model with the most suitable parameters by grid search algorithm; (II) the two-layer LSTM model used in ERGO; (III) the BiLSTM model with the same parameters as model II; (IV) the model II with an attention mechanism; and (V) Attn-BiLSTM, the model III with an attention mechanism. We summarized their performances under the AttnTAP framework with the TPP-I task. The five-fold CV results on McPAS-TCR and VDJdb datasets are shown in Table 4.

**TABLE 4:** The performance of AttnTAP under varied TCR feature extraction models.

		ACC^a^	REC	PRE	F1	AUC
**McPAS** **-TCR**	I^b^	0.736	0.803	0.708	0.752	0.827
	II	0.762	0.803	0.743	0.772	0.854
	III	0.766	0.807	0.747	0.775	0.857
	IV	0.774	0.755	0.755	0.758	0.861
	V	**0.781**	**0.818**	**0.762**	**0.789**	**0.869**
**VDJdb**	I	0.840	0.820	0.855	0.837	0.906
	II	0.839	0.799	0.868	0.832	0.901
	III	0.842	0.806	0.869	0.836	0.904
	IV	0.844	0.820	0.861	0.840	0.908
	V	**0.847**	**0.829**	**0.870**	**0.844**	**0.914**

a Abbreviations: ACC: accuracy; REC: recall; PRE: precision; F1: F1 score; AUC: area under the receiver operating characteristic curve.

b Model numbers: I: the multilayer perceptron model; II: the two-layer long short term memory (LSTM) model; III: the one-layer bi-directional LSTM model; IV: the two-layer LSTM model with attention mechanism; and V: Attn-BiLSTM model.

The results revealed that the BiLSTM model (model III) performed better than the MLP (model I) and LSTM (model II) on the McPAS-TCR dataset, and their three models had similar performance on the VDJdb dataset. The BiLSTM outperformed other feature extraction models without attention mechanism because it considered both precursor and successor amino acids, which extracted information on the interrelationships between amino acids in a more rational way. The models with attention mechanism, especially Attn-BiLSTM (model V), outperformed the other models without attention mechanism in terms of their ACC, REC, PRE, recall, F1 score, and AUC, which indicated that attention algorithms could focus on the key amino acids when processing large amounts of CDR3 information and improve the feature extraction. In AttnTAP, the BiLSTM layer and subsequent attention layer formed the main part of the CDR3 feature extraction model. The attention mechanism assigned various weights to the amino acid features output by the BiLSTM layer, correctly modeling the interrelationships between amino acids and paying more attention to the amino acids that contributed to the antigen-binding specificity (Supplementary Figure S2). As a result, Attn-BiLSTM achieved the highest, and balanced REC (mean 0.818 and 0.829 on McPAS-TCR and VDJdb, respectively) and PRE (mean 0.762 and 0.870 on McPAS-TCR and VDJdb, respectively) on two datasets. Furthermore, the AUC value of Attn-BiLSTM had reached as high as 0.869 and 0.914 on McPAS-TCR and VDJdb. To some extent, the BiLSTM model based on the attention mechanism could improve the performance of TCR-peptide prediction accuracy.

#### 3.1.3 AttnTAP performance on the unbalanced dataset

A real TCR repertoire usually contains more negative samples than positive samples. To validate the performance of the AttnTAP model on an unbalanced dataset and make it suitable for practice, we attempted to generate 14 unbalanced datasets (the ratio of negative to positive samples ranged from 2 to 15) using Supplementary Algorithm S1 in this section. The five-fold CV was used to evaluate the performance of AttnTAP on different unbalanced data (Table 5 and Supplementary Figure S3).

**TABLE 5:** The AUC of AttnTAP on unbalanced datasets.

Ratio	McPAS-TCR	VDJdb	Ratio	McPAS-TCR	VDJdb
1:1^a^	0.838^b^	0.908	1:9	0.865	0.914
1:2	0.853	0.910	1:10	0.870	0.911
1:3	0.854	0.912	1:11	0.872	0.912
1:4	0.863	0.913	1:12	0.873	0.912
1:5	0.862	0.911	1:13	0.872	0.913
1:6	0.871	0.909	1:14	0.870	0.912
1:7	0.867	0.909	1:15	0.868	0.913
1:8	0.870	0.912	-	-	-

a It denotes the ratio of positive samples to negative samples in the dataset.

b We used the metric, area under the receiver operating characteristic curve, to evaluate the performance of the model.

The average AUC on the McPAS-TCR dataset had been rising from 0.838 to 0.873 during the increased number of negative samples, while the average AUC on the VDJdb dataset had reached 0.9 across all the unbalanced data. The AUC performance results indicated that AttnTAP could consistently perform well on unbalanced datasets with an increased number of negative samples.

### 3.2 Performance evaluation of comparative approaches

#### 3.2.1 Performance evaluation of the TPP-I task

According to the requirements of the six deep neural networks (Table 2), we selected only the CDR3 β chains (without the α chains) and discarded the extra amino acids of the sequences longer than the maximum length input. We performed five-fold CVs six times to reduce the unbiased evaluation. We trained the pre-training models of ERGO-LSTM, ERGO-AE, NetTCR-2.0, and AttnTAP, while the pre-training models of ImRex and DLpTCR were downloaded directly (https://github.com/pmoris/ImRex/; https://github.com/jiangBiolab/DLpTCR/) as previously described (Montemurro et al., 2021; Xu et al., 2021). We calculated the scores of five measurements for the different TCR-peptide binding prediction approaches across the two basic datasets. The ACC, REC, PRE, F1, and AUC values, with 95% confidence intervals, for a total of 30 validations experiments, were statistically analyzed (Table 6 and Supplementary Table S2). Briefly, among six prediction approaches, AttnTAP had the highest mean AUC values on both two datasets (the mean values were 0.84 on McPAS-TCR and 0.894 on VDJdb), and the AUC values ranged from 0.824 to 0.860 on McPAS-TCR and ranged from 0.882 to 0.905 on VDJdb (Supplementary Table S2). Moreover, AttnTAP outperformed all other methods overall with respect to the other four metrics, where, in particular, the REC and PRE of its prediction results on the datasets were balanced, indicating its good robustness and stability. Therefore, the AttnTAP was an optimal framework for predicting a TCR-peptide binding.

**TABLE 6:** The performance evaluation of TPP-I task.

		ACC^a^ ^,^ ^b^	REC	PRE	F1	AUC
**McPAS** **-TCR**	ERGO-LSTM	0.748 ± 0.004	0.747 ± 0.013	0.748 ± 0.007	0.747 ± 0.006	0.831 ± 0.005
	ERGO-AE	0.734 ± 0.004	0.696 ± 0.020	0.754 ± 0.009	0.722 ± 0.008	0.808 ± 0.004
	ImRex	0.631 ± 0.003	0.625 ± 0.005	0.648 ± 0.005	0.636 ± 0.004	0.694 ± 0.003
	DLpTCR	0.502 ± 0.003	0.500 ± 0.004	**0.861 ± 0.003**	0.633 ± 0.003	0.529 ± 0.004
	NetTCR-2.0	0.728 ± 0.004	0.734 ± 0.010	0.715 ± 0.018	0.722 ± 0.006	0.799 ± 0.004
	AttnTAP	**0.758 ± 0.003**	**0.769 ± 0.013**	0.752 ± 0.007	**0.760 ± 0.005**	**0.840 ± 0.003**
**VDJdb**	ERGO-LSTM	0.834 ± 0.003	0.790 ± 0.004	0.864 ± 0.004	0.825 ± 0.003	0.889 ± 0.003
	ERGO-AE	0.837 ± 0.003	0.798 ± 0.006	0.864 ± 0.006	0.829 ± 0.004	0.891 ± 0.003
	ImRex	0.561 ± 0.004	0.556 ± 0.005	0.571 ± 0.006	0.564 ± 0.005	0.598 ± 0.004
	DLpTCR	0.482 ± 0.005	0.487 ± 0.004	0.861 ± 0.004	0.622 ± 0.004	0.503 ± 0.005
	NetTCR-2.0	0.832 ± 0.003	**0.851 ± 0.008**	0.802 ± 0.007	0.826 ± 0.003	0.890 ± 0.002
	AttnTAP	**0.839 ± 0.003**	0.801 ± 0.006	**0.865 ± 0.004**	**0.831 ± 0.003**	**0.894 ± 0.002**

a The results show 95% confidence intervals for all the validations (totally 30 validations for each cross-validation).

b Abbreviations: ACC: accuracy; REC: recall; PRE: precision; F1: F1 score; AUC: area under the receiver operating characteristic curve.

### 3.2.2 Performance evaluation of the TPP

To further validate the generalization performance of these methods, we evaluated them in the TPP-II task and conducted independent replicate experiments 30 times. Similar to the TPP-I task, the AttnTAP model achieved the highest AUC values (the mean values were 0.837 on McPAS-TCR and 0.893 on VDJdb) (Table 7), and the AUC values ranged from 0.810 to 0.864 on McPAS-TCR and ranged from 0.873 to 0.908 on VDJdb in the TPP-II task (Supplementary Table S3). Moreover, it had better overall performance than other methods in terms of the other four metrics, with a balanced REC and PRE. As a result, compared with the existing methods, AttnTAP had better generalization and could perform better on new data.

**TABLE 7:** The performance evaluation of TPP-II task.

		ACC^a^ ^,^ ^b^	REC	PRE	F1	AUC
**McPAS** **-TCR**	ERGO-LSTM	0.735 ± 0.005	0.761 ± 0.016	0.724 ± 0.009	0.741 ± 0.006	0.818 ± 0.004
	ERGO-AE	0.731 ± 0.005	0.672 ± 0.022	0.764 ± 0.012	0.712 ± 0.009	0.800 ± 0.005
	ImRex	0.627 ± 0.004	0.621 ± 0.006	0.644 ± 0.007	0.632 ± 0.005	0.690 ± 0.005
	DLpTCR	0.501 ± 0.003	0.499 ± 0.003	**0.859 ± 0.004**	0.631 ± 0.003	0.524 ± 0.004
	NetTCR-2.0	0.731 ± 0.004	0.746 ± 0.008	0.699 ± 0.018	0.720 ± 0.008	0.804 ± 0.004
	AttnTAP	**0.755 ± 0.005**	**0.778 ± 0.011**	0.743 ± 0.006	**0.760 ± 0.006**	**0.837 ± 0.004**
**VDJdb**	ERGO-LSTM	0.832 ± 0.003	0.794 ± 0.007	0.860 ± 0.005	0.825 ± 0.004	0.891 ± 0.003
	ERGO-AE	0.836 ± 0.003	0.800 ± 0.009	0.864 ± 0.005	0.830 ± 0.004	0.888 ± 0.004
	ImRex	0.561 ± 0.005	0.560 ± 0.006	0.575 ± 0.006	0.568 ± 0.006	0.597 ± 0.006
	DLpTCR	0.488 ± 0.004	0.494 ± 0.004	0.862 ± 0.004	0.628 ± 0.004	0.510 ± 0.004
	NetTCR-2.0	0.832 ± 0.003	**0.860 ± 0.007**	0.794 ± 0.009	0.825 ± 0.004	0.891 ± 0.003
	AttnTAP	**0.838 ± 0.003**	0.794 ± 0.006	**0.872 ± 0.004**	**0.831 ± 0.004**	**0.893 ± 0.003**

a The results show 95% confidence intervals for totally 30 independent experiments.

b Abbreviations: ACC: accuracy; REC: recall; PRE: precision; F1: F1 score; AUC: area under the receiver operating characteristic curve.

### 3.2.3 Computational costs of approaches

**FIGURE 3:**
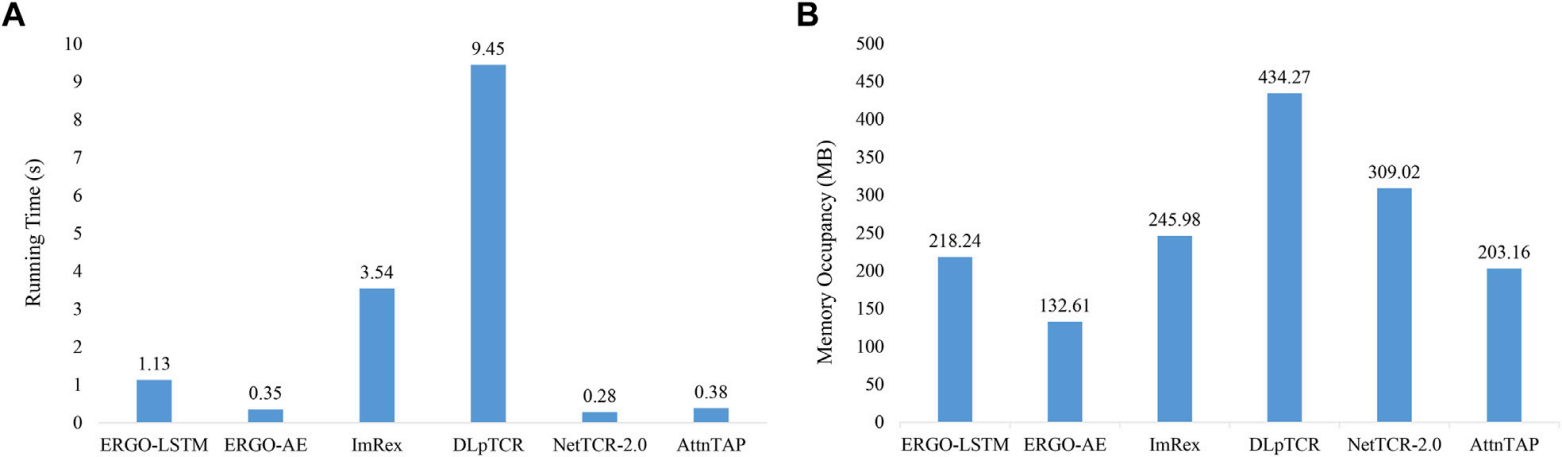
The computational costs of the approaches on the McPAS-TCR dataset. **(A)** The average running time of the six approaches on the McPAS-TCR dataset in the TPP-I task. **(B)** The required memory occupancy of the six approaches on the McPAS-TCR dataset in the TPP-I task.

In this study, the average running time was recorded 30 times independent experiments (Figure 3A and Supplementary Table S2). Figure 3A demonstrates that NetTCR-2.0, ERGO-AE, and AttnTAP had similar running times, which was much less than the other three approaches, while DLpTCR achieved the longest running time, which indicated that DLpTCR had a higher complexity of model configuration. The required memory occupancy of all the six approaches on the McPAS-TCR datasets was also recorded and averaged for comparison (Figure 3B and Supplementary Table S2). The running ERGO-AE with the minimal space and followed by AttnTAP, whereas the DLpTCR had the largest space occupancy for its complex framework. Thus, AttnTAP improved the accuracy of TCR-peptide binding prediction while being quite efficient in terms of computational time and memory usage.

## 4 Discussion

The prediction of TCRs binding to the peptide is urgent in a clinical, but still extremely challenging, with highly cross-reactive TCRs and peptides, unseen peptides lack biological verification, and limited available training samples (Rudolph et al., 2006; Szeto et al., 2020; Moris et al., 2021). The breakthrough of deep convolutional neural networks in predicting TCR-peptide binding accuracy, accelerating well-integrated human immune repertoire, and potentially interacting peptides prediction pipelines. However, a few remaining issues led us to design this experiment. In this study, we designed the attention mechanism under the Attn-BiLSTM framework, considering the various contributions of amino acids in CDR3 sequences. Then, a dual input of CDR3 sequences and peptides was needed to improve the prediction accuracy, instead of separate embedding steps ignoring the two protein molecular interactors. The experimental results also showed the AttnTAP achieved a good performance in TCR-peptide binding prediction.

Due to the high dimensionality, non-homogeneous, and sparsity of TCR repertoire data, we proposed a novel and unified architecture, which combined a bi-directional LSTM (BiLSTM), an attention mechanism, and a convolutional layer. The BiLSTM extracted TCR features by considering both the preceding and succeeding amino acid representations of a single CDR3 chain (Zhou et al., 2016). Moreover, an attention mechanism was employed to give a different focus to the information outputted from the hidden layers of BiLSTM. In Supplementary Figure S2, the weight of amino acids in a CDR3 chain varies greatly at different positions, with the color changed from light to dark. It is a biological truism that high weights (dark) tend to appear in the middle region of a CDR3 chain (Robins et al., 2009), and the weighting pattern displayed by AttnTAP on most CDR3 sequences was consistent with this truism. However, some sequences had special weighting patterns, showing strong weighting at the beginning or ending amino acids (N- or C- terminus of the CDR loop). We analyzed the attention weight condition of 1957 test samples from the VDJdb dataset in one five-fold CV test. We found that AttnTAP exhibited strong weighting for their beginning part only on 59 CDR3 sequences, which represented only 0.03 of all the samples, and these sequences corresponded to 31 different peptides. Furthermore, some CDR3 sequences showed strong weighting at the terminal amino acids (C- terminus) of the shorter sequences as well as the placeholders. Given that the attention mechanism may assign higher weights to the boundary part, where the anterior and posterior position features differ, AttnTAP focused on the terminal amino acid “F” and the placeholders, taking into account the sequence length feature. In addition, we also speculated that some CDR3 sequences had unexpected patterns due to the strong V or J region preferences or the dataset biases. Although most CDR3 sequences have a similar beginning or ending (e.g., beginning with “C” and ending with “F”), these similar beginnings and endings may still form specific combinations with highly variable amino acids in the middle of the sequences, which allows the sequences to possess antigen-binding specificity.

**FIGURE 4:**
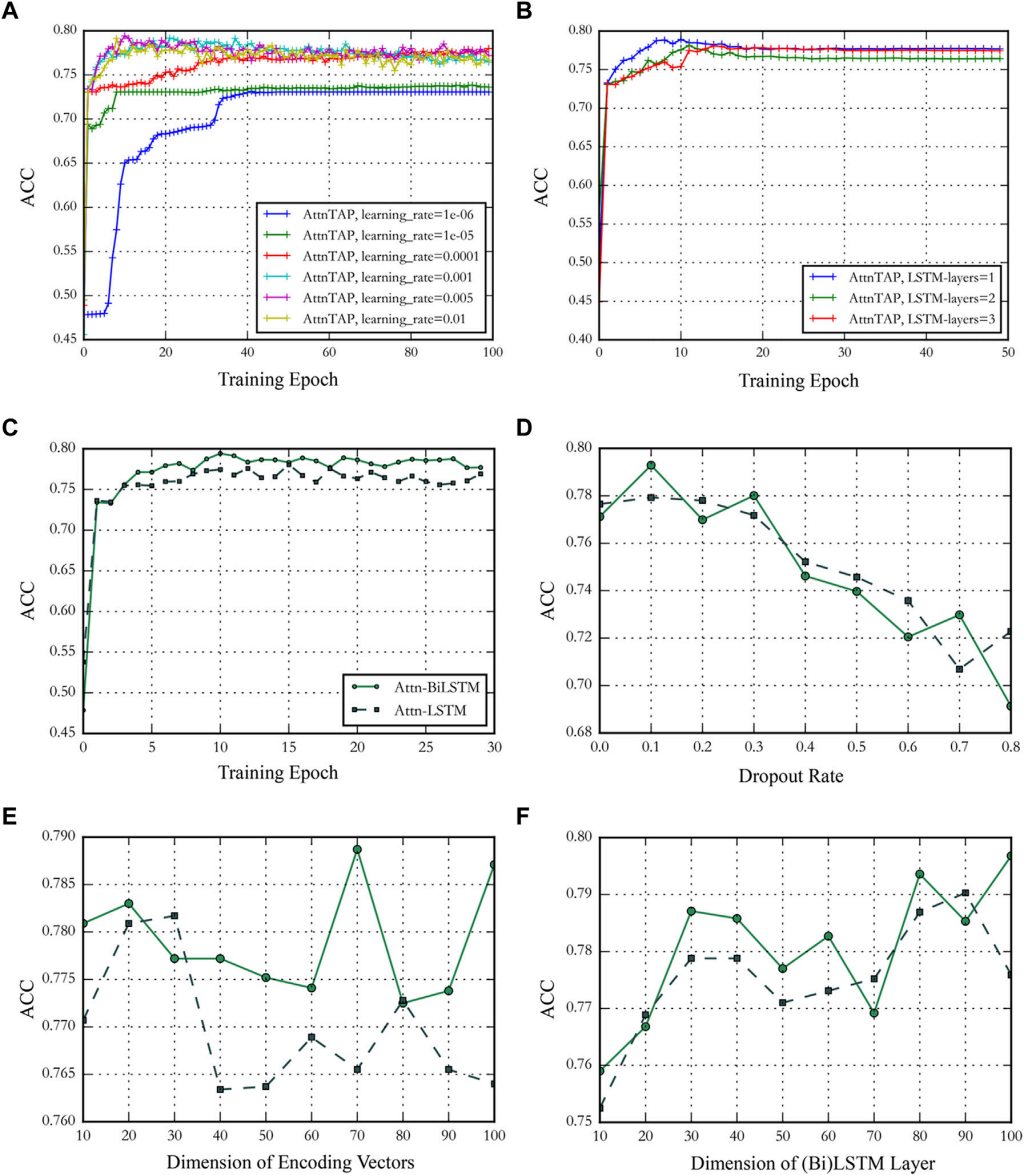
The performance of AttnTAP with different hyperparameters. **(A,B)** Panels showed the performance of AttnTAP with different learning rates and bi-directional long short-term (BiLSTM) layer numbers. **(C–F)** Panels depicted the performance of AttnTAP using LSTM/BiLSTM with different training epochs, dropout rates, dimensions of encoding vectors, and LSTM/BiLSTM layers, respectively.

As is well-known, an adjustable hyperparameter, including the learning rate, the number of BiLSTM layers, the training epoch, and the dropout rate, could balance the latent channel capacity and improve the prediction accuracy (Graves et al., 2013; Zhou et al., 2016). We conducted a series of experiments on the McPAS-TCR dataset to validate the effect of different hyperparameters on model prediction performances and determine the value of the hyperparameters based on the results. We used the metric ACC to evaluate the model prediction accuracy in the experiments (Figure 4). Four hyperparameters, including training epoch, dropout rate, dimension of encoding vectors, and the dimension of LSTM/BiLSTM layers, were used to compare the performance of Attn-LSTM and Attn-BiLSTM (Figures 4C–F). BiLSTM was an ideal model under the different hyperparameters conditions. Thus, in this study, we set the training epoch, the dropout rate, the dimensions of amino acid encoding vectors, and the BiLSTM layer to 10, 0.1, 70, and 80 for AttnTAP, respectively, according to the results. The ACC had deteriorated significantly when the learning rate was below 0.0001, thus we set the threshold to 0.001 for compatibility with the application in the various dataset (Figure 4A). There was no significant improvement in model performance as the number of BiLSTM layers increased, we used one-layer BiLSTM to reduce model complexity (Figure 4B).

ImRex and DLpTCR had lower prediction accuracies than the other four approaches under TPP-I and TPP-II tasks, maybe due to the overfitting caused by their complex model structures. We reduced the complexity of AttnTAP by using one-layer BiLSTM instead of multi-layer BiLSTM to extract TCR sequences features and the MLP model instead of the LSTM model to extract peptide features to avoid the overfitting. The results of AttnTAP in TPP-II were similar to those in TPP-I, which indicated that AttnTAP had a robust and good generalization in predicting an unseen TCR sequence binding to a peptide. Thus, the AttnTAP presented here could serve as an unseen TCR-peptide prediction method, for accelerating identifying neoantigens and activated T cells for immunotherapy clinically.

**FIGURE 5:**
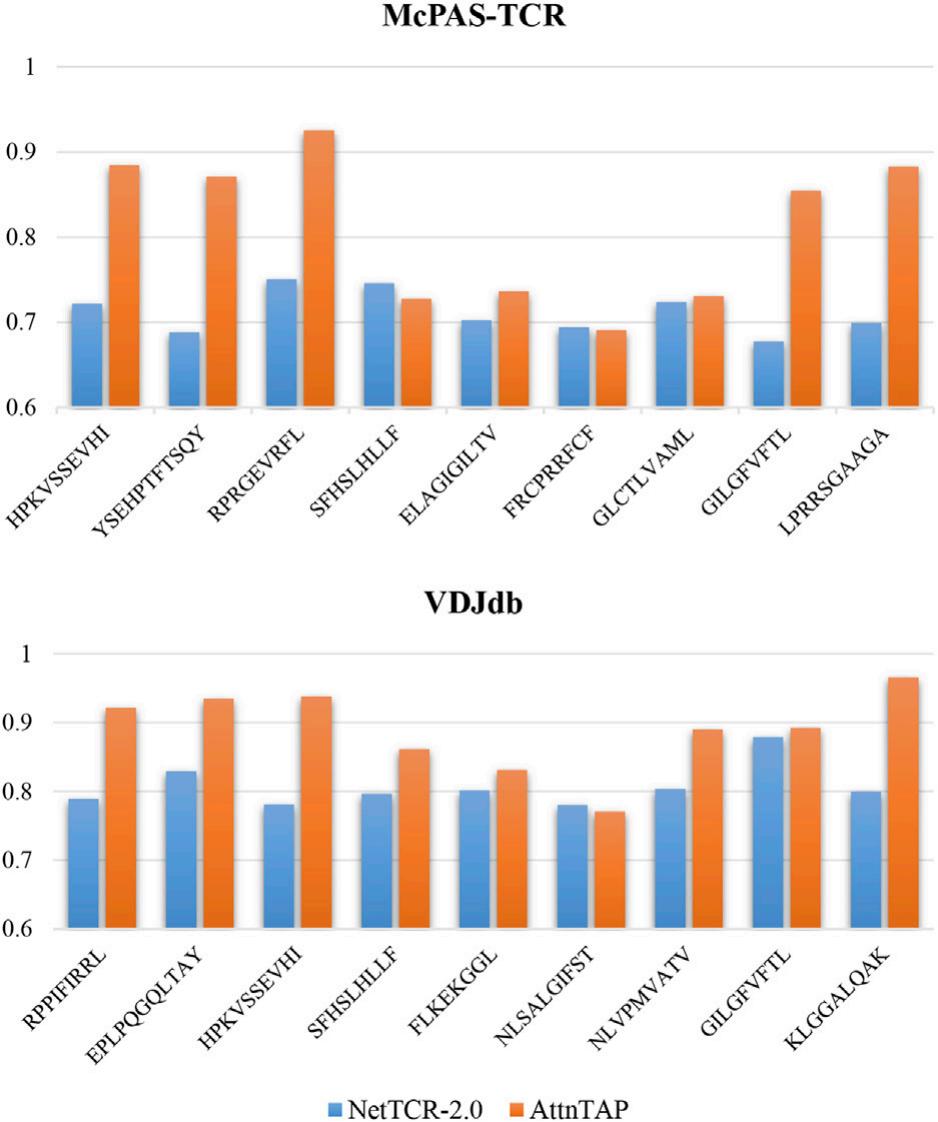
The prediction performance of AttnTAP and NetTCR-2.0 on different peptides. We first selected nine peptides according to abundance for the McPAS-TCR and VDJdb datasets, respectively, of which three were of lowest abundance (McPAS-TCR: HPKVSSEVHI, YSEHPTFTSQY, RPRGEVRFL; VDJdb: RPPIFIRRL, EPLPQGQLTAY, HPKVSSEVHI), three were of intermediate abundance (McPAS-TCR: SFHSLHLLF, ELAGIGILTV, FRCPRRFCF; VDJdb: SFHSLHLLF, FLKEKGGL, NLSALGIFST), and three were of highest abundance (McPAS-TCR: GLCTLVAML, GILGFVFTL, LPRRSGAAGA; VDJdb: NLVPMVATV, GILGFVFTL, KLGGALQAK). Then, we performed five-fold cross-validation on NetTCR-2.0 and AttnTAP using only TCR β chain CDR3 sequences, where we used all training data to train the models but only used the test data containing the corresponding peptide to test the models. The average accuracy was used to evaluate their prediction performance.

In addition to the performances of AttnTAP on the entire McPAS-TCR and VDJdb datasets, we also evaluated its performances on different peptides, especially the peptides with low abundance, in the TPP-I task. The abundance of peptides in the McPAS-TCR dataset ranged from 0.005 to 0.219, and from 0.001 to 0.356 in the VDJdb dataset (Supplementary Table S1). We selected nine peptides according to their abundances (high-, medium- and low-abundance accounted for one-third) for the McPAS-TCR and VDJdb datasets, respectively (Supplementary Table S1). Considering that NetTCR-2.0 is the latest method for TCR-peptide binding prediction and has high prediction accuracies with low computational cost, we selected it as the baseline model. We performed a five-fold CV on NetTCR-2.0 and AttnTAP using only TCR β chain CDR3 sequences and compared their performance by average ACC. In detail, we used all training data to train the models, while only used the test data containing the corresponding peptide to test the models (Figure 5 and Supplementary Table S4). On the McPAS-TCR dataset, the average ACCs of AttnTAP and NetTCR-2.0 were 0.894 and 0.720 for the lowest abundance peptides, 0.718 and 0.714 for the intermediate abundance peptides, and 0.823 and 0.700 for the highest abundance peptides. Moreover, on the VDJdb dataset, their average ACCs were 0.932 and 0.800 for the lowest abundance peptides, 0.821 and 0.793 for the intermediate abundance peptides, and 0.916 and 0.828 for the highest abundance peptides, respectively. The results indicated that AttnTAP had higher ACCs than NetTCR-2.0 on most of the peptides and had similar performances to the latter on the other peptides (e.g., SFHSLHLLF and FRCPRRFCF in the McPAS-TCR dataset and NLSALGIFST in the VDJdb dataset). In our opinion, the AttnTAP framework had a good performance on TCR-peptide binding prediction, especially the low-abundance peptides, due to its BiLSTM model with attention mechanism in extracting CDR3 features, which validated that AttnTAP has good stability and robustness.

In conclusion, we successfully trained a dual-input model to predict the interactions between seen and unseen TCRs and peptides. Due to the limited training samples and known peptides we had available, we tried to reduce the complexity of the model to avoid overfitting on the premise of prediction accuracy. In the future, we will consider more information on TCR sequences, such as the CDR1 and CDR2, or TCRα chain when data become available, to train a good performance and more generalization prediction model to be suitable for multi-types data, meeting the urgent clinical needs.

## Data Availability

AttnTAP is available on GitHub, at https://github.com/Bioinformatics7181/AttnTAP/, for academic use only. The publicly available data for this study can be found in VDJdb (https://vdjdb.cdr3.net/), IEDB (http://www.iedb.org/), and McPAS-TCR (http://friedmanlab.weizmann.ac.il/McPAS-TCR/). The pre-training models of ImRex and DLpTCR can be found on Github (https://github.com/pmoris/ImRex/; https://github.com/jiangBiolab/DLpTCR/); further inquiries can be directed to the corresponding author.

## Supplementary material

The Supplementary Material for this study can be found online at: https://www.frontiersin.org/articles/10.3389/fgene.2022.942491/full#supplementary-material

## References

[B1] Asgari E. Mofrad M. (2015). Continuous distributed representation ofbiological sequences for deep proteomics and genomics. PLoS One 10 (11), e0141287. 10.1371/journal.pone.0141287 26555596 PMC4640716

[B2] Bagaev D. V. Vroomans R. M. A. Samir J. Stervbo U. Rius C. Dolton G. (2020). VDJdb in 2019: Database extension, new analysis infrastructure and a T-cell receptor motif compendium. Nucleic Acids Res. 48 (D1), D1057–D1062. 10.1093/nar/gkz874 31588507 PMC6943061

[B3] Bahdanau D. Cho K. Bengio Y. (2014). Neural machine translation by jointly learning to align and translate. *arXiv* . 10.48550/arXiv.1409.0473

[B4] Bolotin D. A. Mamedov I. Z. Britanova O. V. Zvyagin I. V. Shagin D. Ustyugova S. V. (2012). Next generation sequencing for TCR repertoire profiling: Platform-specific features and correction algorithms. Eur. J. Immunol. 42 (11), 3073–3083. 10.1002/eji.201242517 22806588

[B5] Chiffelle J. Genolet R. Perez M. A. Coukos G. Zoete V. Harari A. (2020). T-cell repertoire analysis and metrics of diversity and clonality. Curr. Opin. Biotechnol. 65, 284–295. 10.1016/j.copbio.2020.07.010 32889231

[B6] Crooks G. Hon G. Chandonia J. M. Brenner S. E. (2004). Weblogo: A sequence logo generator. Genome Res. 14 (6), 1188–1190. 10.1101/gr.849004 15173120 PMC419797

[B7] Glanville J. Huang H. Nau A. Hatton O. Wagar L. E. Rubelt F. (2017). Identifying specificity groups in the T cell receptor repertoire. Nature 547 (7661), 94–98. 10.1038/nature22976 28636589 PMC5794212

[B8] Graves A. Mohamed A. R. Hinton G. (2013). “Speech recognition with deep recurrent neural networks,”in 2013 IEEE International Conference on Acoustics, Speech and Signal Processing, Vancouver, BC, Canada, 26-31 May 2013 (IEEE), 6645–6649. 10.48550/arXiv.1303.5778

[B9] Henikoff S. Henikoff J. G. (1992). Amino acid substitution matrices from protein blocks. Proc. Natl. Acad. Sci. U. S. A. 89, 10915–10919. 10.1073/pnas.89.22.10915 1438297 PMC50453

[B10] Joglekar A. V. Li G. (2021). T cell antigen discovery. Nat. Methods 18 (8), 873–880. 10.1038/s41592-020-0867-z 32632239

[B11] La Gruta N. L. Gras S. Daley S. R. Thomas P. G. Rossjohn J. (2018). Understanding the drivers of MHC restriction of T cell receptors. Nat. Rev. Immunol. 18 (7), 467–478. 10.1038/s41577-018-0007-5 29636542

[B12] LeCun Y. Bengio Y. Hinton G. (2015). Deep learning. Nature 521 (7553), 436–444. 10.1038/nature14539 26017442

[B13] Mahajan S. Vita R. Shackelford D. Lane J. Schulten V. Zarebski L. (2018). Epitope specific antibodies and T cell receptors in the immune epitope database. Front. Immunol. 9, 2688. 10.3389/fimmu.2018.02688 30515166 PMC6255941

[B14] Mikolov T. Chen K. Corrado G. Dean J. (2013). Efficient estimation of word representations in vector space. *arXiv* . 10.48550/arXiv.1301.3781

[B15] Montemurro A. Schuster V. Povlsen H. R. Bentzen A. K. Jurtz V. Chronister W. D. (2021). NetTCR-2.0 enables accurate prediction of TCR-peptide binding by using paired TCRα and β sequence data. Commun. Biol. 4 (1), 1060. 10.1038/s42003-021-02610-3 34508155 PMC8433451

[B16] Moris P. De Pauw J. Postovskaya A. Gielis S. De Neuter N. Bittremieux W. (2021). Current challenges for unseen-epitope TCR interaction prediction and a new perspective derived from image classification. Brief. Bioinform. 22 (4), bbaa318. 10.1093/bib/bbaa318 33346826 PMC8294552

[B17] Robins H. S. Campregher P. V. Srivastava S. K. Wacher A. Turtle C. J. Kahsai O. (2009). Comprehensive assessment of T-cell receptor beta-chain diversity in alphabeta T cells. Blood 114 (19), 4099–4107. 10.1182/blood-2009-04-217604 19706884 PMC2774550

[B18] Rudolph M. G. Stanfield R. L. Wilson I. A. (2006). How TCRs bind MHCs, peptides, and coreceptors. Annu. Rev. Immunol. 24, 419–466. 10.1146/annurev.immunol.23.021704.115658 16551255

[B19] Schneider T. Stephens R. (2002). Sequence logos: A new way to display consensus sequences. Nucleic Acids Res. 18 (20), 6097–6100. 10.1093/nar/18.20.6097 PMC332411 2172928

[B20] Springer I. Besser H. Tickotsky-Moskovitz N. Dvorkin S. Louzoun Y. (2020). Prediction of specific TCR-peptide binding from large dictionaries of TCR-peptide pairs. Front. Immunol. 11, 1803. 10.3389/fimmu.2020.01803 32983088 PMC7477042

[B21] Srivastava N. Hinton G. Krizhevsky A. Sutskever I. Salakhutdinov R. (2014). Dropout: A simple way to prevent neural networks from overfitting. J. Mach. Learn. Res. 15 (1), 1929–1958.

[B22] Szeto C. Lobos C. A. Nguyen A. T. Gras S. (2020). TCR recognition of peptide-MHC-I: Rule makers and breakers. Int. J. Mol. Sci. 22 (1), 68. 10.3390/ijms22010068 PMC7793522 33374673

[B23] Tickotsky N. Sagiv T. Prilusky J. Shifrut E. Friedman N. (2017). McPAS-TCR: A manually curated catalogue of pathology-associated T cell receptor sequences. Bioinformatics 33 (18), 2924–2929. 10.1093/bioinformatics/btx286 28481982

[B24] Tran N. H. Xu J. Li M. (2022). A tale of solving two computational challenges in protein science: Neoantigen prediction and protein structure prediction. Brief. Bioinform. 23 (1), bbab493. 10.1093/bib/bbab493 34891158 PMC8769896

[B25] Vaswani A. Shazeer N. Parmar N. Uszkoreit J. Jones L. Gomez A. N. (2017). Attention is all you need. Adv. Neural Inf. Process. Syst. 3030 (NIPS), 1–15. 10.48550/arXiv.1706.03762

[B26] Wagih O. (2017). Ggseqlogo: A versatile r package for drawing sequence logos. Bioinformatics 33 (22), 3645–3647. 10.1093/bioinformatics/btx469 29036507

[B27] Warren R. L. Freeman J. D. Zeng T. Choe G. Munro S. Moore R. (2011). Exhaustive T-cell repertoire sequencing of human peripheral blood samples reveals signatures of antigen selection and a directly measured repertoire size of at least 1 million clonotypes. Genome Res. 21 (5), 790–797. 10.1101/gr.115428.110 21349924 PMC3083096

[B28] Woodsworth D. J. Castellarin M. Holt R. A. (2013). Sequence analysis of T-cell repertoires in health and disease. Genome Med. 5 (10), 98. 10.1186/gm502 24172704 PMC3979016

[B29] Xu Z. Luo M. Lin W. Xue G. Wang P. Jin X. (2021). DLpTCR: An ensemble deep learning framework for predicting immunogenic peptide recognized by T cell receptor. Brief. Bioinform. 22, 1–13. 10.1093/bib/bbab335 34415016

[B33] Xu Y. Qian X. Zhang X. Lai X. Liu Y. Wang J. (2022). DeepLION: Deep Multi-Instance Learning Improves the Prediction of Cancer-Associated T Cell Receptors for Accurate Cancer Detection. Front. Genet. 13:860510. 10.3389/fgene.2022.860510 PMC9121378 35601486

[B30] Zemouri R. Zerhouni N. Racoceanu D. (2019). Deep learning in the biomedical applications: Recent and future status. Appl. Sci. (Basel). 9 (8), 1526. 10.3390/app9081526

[B31] Zhao L. Liu H. Yuan X. Gao K. Duan J. (2020). Comparative study of whole exome sequencing-based copy number variation detection tools. BMC Bioinforma. 21 (1), 97. 10.1186/s12859-020-3421-1 PMC7059689 32138645

[B32] Zhou P. Shi W. Tian J. Qi Z. Li B. Hao H. (2016). Attention-based bidirectional long short-term memory networks for relation classification. Proc. 54th Annu. Meet. Assoc. Comput. Linguistics 2, 207–212. 10.18653/v1/P16-2034

