# Supplementary material for: AttnTAP: A Dual-input Framework Incorporating the Attention Mechanism for Accurately Predicting TCR-peptide Binding
Source: Front Genet. 2022 Aug 22;13:942491. doi: 10.3389/fgene.2022.942491 (PMC9441555; doi:10.3389/fgene.2022.942491)
Supplement: Supplementary file 2 [file DataSheet1.docx]

Supplementary Material

# Supplementary Algorithm

| **Input:** D = {(*t_i_*, *a_i_*) \| *i* = 1, 2, …, *n*}, sampleNumbers, randomSeed |
| --- |
| **Output:** negSamples = {(*t_i_*, *a_i_*, *y_i_*) \| *i* = 1, 2, …, *m*} |
| **1** epitopeSet = getEpitopeSet(D) // Get all epitopes |
| **2** negSamples = [] // Initialize the negative sample list |
| **3 for** *i* = 0, …, len(epitopeSet) - 1 **do** |
| **4**  epitopeList = removeMatchedEpitope (epitopeSet, *i*) |
| **5 for** *j* = 0, …, len(D) - 1 **do** |
| **6**  sequence = D[*j*][0] // Get the (*j*+1)th CDR3 sequence |
| **7 for** *k* = 0, …, sampleNumbers - 1 **do** |
| **8**  random = random(randomSeed) // Set random numbers |
| **9**  negEpitope = random.choice(epitopeList) // Select epitopes randomly |
| **10** negSamples.append([sequence, negEpitope, 0]) // Add the pairs to result list |
| **11 end** |
| **12 end** |
| **13 end** |

**Supplementary Algorithm 1.** The procedure for generating negative samples

# Supplementary Figures


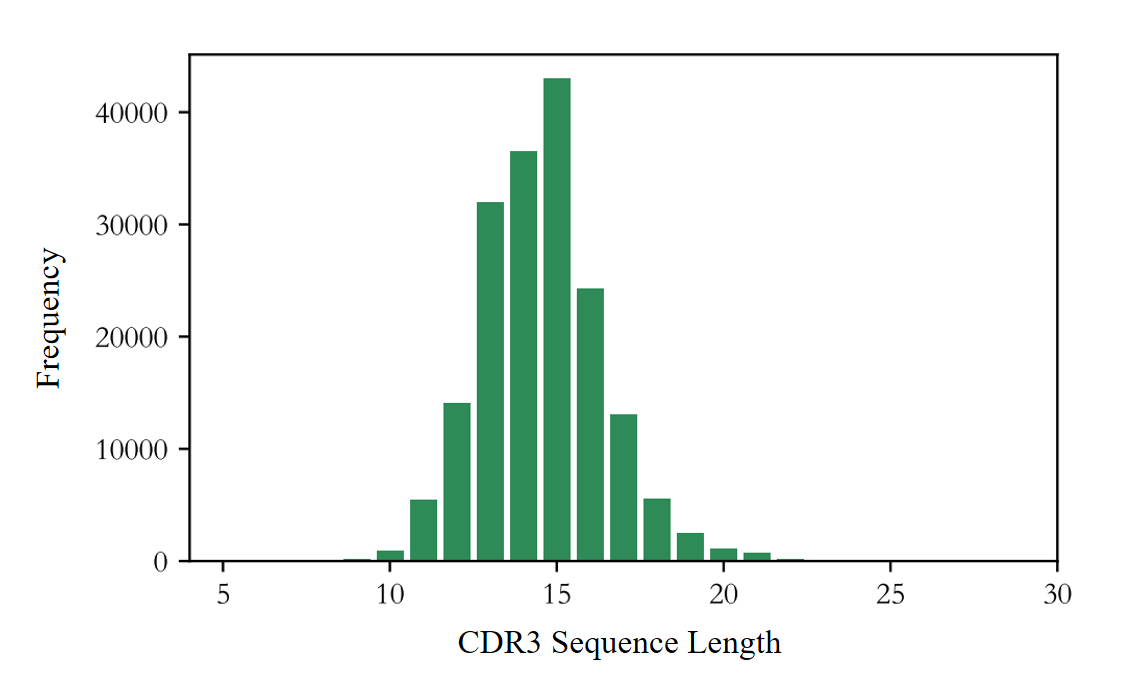


**Supplementary Figure 1.** The frequency statistic of the CDR3β sequences in length. The 181,436 CDR3β sequences obtained from IDEB, McPAS-TCR and VDJdb datasets range in length from 6 to 27 amino acids, with the majority falling between 11 and 18.


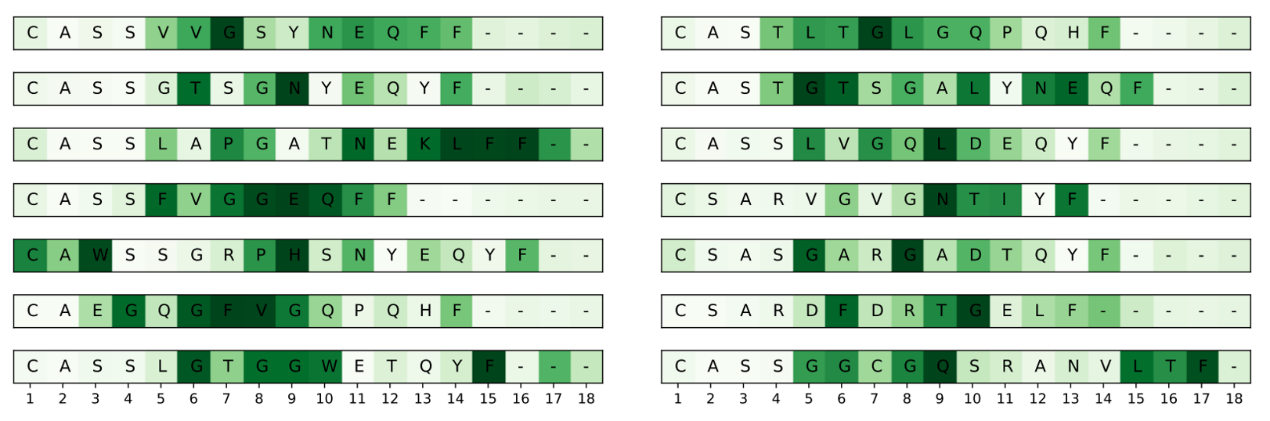


**Supplementary Figure 2.** The weights assigned to each amino acid position by attention mechanism. The darker the color of an amino acid, the more weight the model gives to that position.


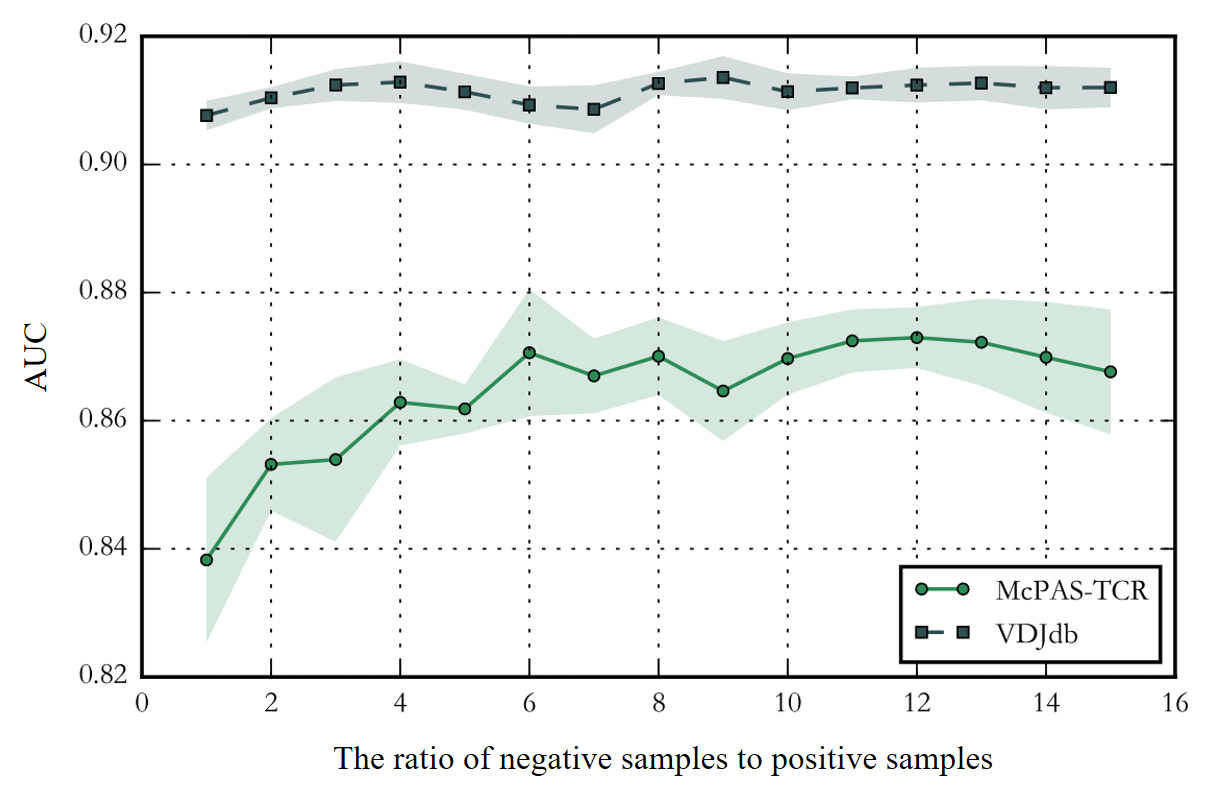


**Supplementary Figure 3.** The AUCs of AttnTAP on the datasets with different ratios. The solid green line represents the average AUCs on the McPAS-TCR dataset, the dashed dark green line represents the average AUCs on the VDJdb dataset, and the shading around the curves represents the standard deviations of the five-fold CV, the area of which can reflect approach's stability across different data divisions.
